# Early Genotoxic and Cytotoxic Effects of the Toxic Dinoflagellate *Prorocentrum lima* in the Mussel *Mytilus galloprovincialis*

**DOI:** 10.3390/toxins8060159

**Published:** 2016-05-24

**Authors:** María Verónica Prego-Faraldo, Vanessa Valdiglesias, Blanca Laffon, Josefina Mendez, Jose M. Eirin-Lopez

**Affiliations:** 1 Chromatin Structure and Evolution Group (Chromevol), Department of Biological Sciences, Florida International University, North Miami, FL 33181, USA; mveronicaprego@gmail.com; 2 XENOMAR Group, Department of Cellular and Molecular Biology, Universidade da Coruña, A Coruna E15071, Spain; fina@udc.es; 3 DICOMOSA Group, Department of Psychology, Area of Psychobiology, Universidade da Coruña, A Coruna E15071, Spain; vvaldiglesias@udc.es (V.V.); blaffon@udc.es (B.L.)

**Keywords:** bivalve molluscs, DSP toxins, okadaic acid, DNA damage, oxidative DNA damage, cytotoxicity, comet assay, flow cytometry, OGG1 enzyme

## Abstract

Okadaic acid (OA) and dinophysistoxins (DTXs) are the main toxins responsible for diarrhetic shellfish poisoning (DSP) intoxications during harmful algal blooms (HABs). Although the genotoxic and cytotoxic responses to OA have been evaluated *in vitro*, the *in vivo* effects of these toxins have not yet been fully explored. The present work fills this gap by evaluating the *in vivo* effects of the exposure to the DSP-toxin-producing dinoflagellate *Prorocentrum lima* during the simulation of an early HAB episode in the mussel *Mytilus galloprovincialis*. The obtained results revealed that *in vivo* exposure to this toxic microalgae induced early genotoxicity in hemocytes, as a consequence of oxidative DNA damage. In addition, the DNA damage observed in gill cells seems to be mainly influenced by exposure time and *P. lima* concentration, similarly to the case of the oxidative damage found in hemocytes exposed *in vitro* to OA. In both cell types, the absence of DNA damage at low toxin concentrations is consistent with the notion suggesting that this level of toxicity does not disturb the antioxidant balance. Lastly, *in vivo* exposure to growing *P. lima* cell densities increased apoptosis but not necrosis, probably due to the presence of a high number of protein apoptosis inhibitors in molluscs. Overall, this work sheds light into the *in vivo* genotoxic and cytotoxic effects of *P. lima*. In doing so, it also demonstrates for the first time the potential of the modified (OGG1) comet assay for assessing oxidative DNA damage caused by marine toxins in marine invertebrates.

## 1. Introduction

Harmful Algal Blooms (HABs) constitute a major environmental threat for marine organisms and human consumers of shellfish. During the last few years, HABs have been displaying increased frequencies and intensities [1,2]. Okadaic acid (OA) and its derivatives, the dinophysistoxins (DTXs), constitute the main lipophilic toxins produced during HABs in the Atlantic coast of Europe [3]. During these episodes, large amounts of these toxins are produced by dinoflagellates from the genera *Dinophysis* and *Prorocentrum*. Their subsequent accumulation by marine organisms is responsible for the Diarrhetic Shellfish Poisoning (DSP) syndrome [4], a disorder causing vomiting, diarrhea, and abdominal pains, among other symptoms, in human consumers of contaminated shellfish [5]. In order to prevent intoxications, the European Union has limited the harvesting and sale of shellfish with OA levels above 160 µg of OA equivalent/kg dry weight (EC 853/2004). Overall, HABs impact aquaculture [6] and the economy of coastal areas, especially those that are heavily dependent on this industry [3,7].

The harmful effects of DSP toxins on human health have motivated studies addressing the mechanisms of action of these molecules, notably in the case of OA and its inhibitory activity on serine/threonine protein phosphatases [8]. Additionally, it has been reported that OA derivatives (*i.e.*, acyl derivatives) may lead to toxicity without actually binding protein phosphatases, although little is known about their effects and synergistic interactions [9]. OA causes alterations on the DNA molecule, on cellular components, on the immune and nervous systems, as well as on the embryonic development of mammalian cells [10]. Studies conducted in invertebrates, more specifically in bivalve molluscs, have suggested that these organisms are able to accumulate high concentrations of DSP toxins thanks to dedicated resistance and detoxification mechanisms. Indeed, while low levels of OA appear to produce an early cytogenotoxic response, the cytogenetic integrity of some cell types recovers rapidly after exposure to high and persistent concentrations of this toxin [11,12,13].

The number of studies addressing the biological responses of marine invertebrates to DSP toxins has increased dramatically over the last 10 years, however, most of them rely on *in vitro* approaches for toxicity assessment (*i.e.*, exposure of organisms to purified individual toxins [11,13]), as these often increase the speed, precision, and reproducibility of analyses while reducing costs [14,15]. On the contrary, the number of *in vivo* studies using complex toxin mixtures are still scarce, including studies considering *Prorocentrum lima*, the dinoflagellate most commonly used as a source of DSP toxins [11,16]. These are particularly interesting because they allow the potential to determine the indirect effects of toxins, improving the assessment of the different types of organismal responses including absorption, distribution, metabolism, and depuration [17]. Although certain studies have observed a correlation between both assays [18], the extrapolation of *in vitro* data to *in vivo* situations is often problematic [19]. Thus, the most realistic way to evaluate the synergistic effects of all toxins involved in HABs episodes would optimally involve the combination of both approaches.

The present work builds on this knowledge to investigate the genotoxic and cytotoxic responses of the mussel *Mytilus galloprovincialis* to low densities of the toxic dinoflagellate *P. lima*, a producer of DSP toxins. To this end, DNA damage was evaluated on hemolymph and gill cells using alkaline comet assay. Hemocytes (based on their low basal damage and easy individualization) were also used to assess the oxidative DNA damage and cytotoxic effects of these toxins using the modified (OGG1) comet assay and flow cytometry, respectively. The obtained results revealed, for the first time, the dynamics of the genotoxic and cytotoxic damage resulting from the *in vivo* exposure to low densities of *P. lima* in marine invertebrates. In doing so, this work pioneers the use of the modified (OGG1) comet assay as a valid experimental approach improving the evaluation of the oxidative DNA damage caused by marine toxins in the hemolymph of marine invertebrates.

## 2. Results

### 2.1. Toxin Accumulation and DNA Damage Resulting from In Vivo Exposure to P. lima

**Figure 1:**
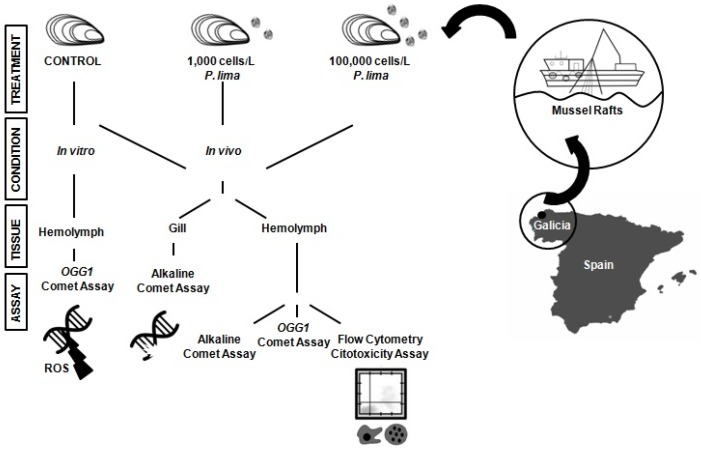
Schematic diagram describing the experimental design followed in the present work. Mussel specimens were collected and acclimated to laboratory conditions before exposing them *in vivo* to different cellular densities of the diarrhetic shellfish poisoning (DSP)-producing dinoflagellate *P. lima* for 24 h and 48 h. The genotoxic and cytotoxic effects of the exposure to *P. lima* were evaluated by means of the comet assay (alkaline and OGG1-modified) and flow cytometry in different cell types. A group of mussels was also exposed *in vitro* to okadaic acid (OA) to quantify oxidative DNA damage in hemolymph cells using the OGG1-modified comet assay.

**Figure 2:**
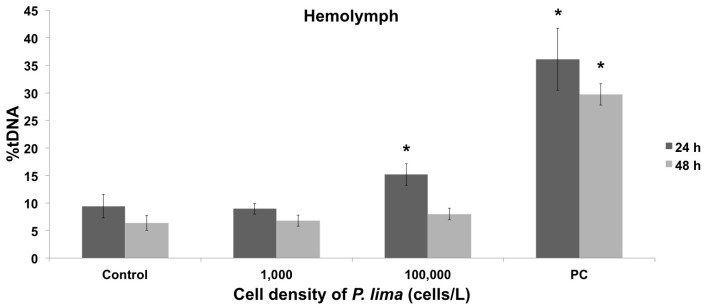
Quantification of DNA damage using the alkaline comet assay in mussel hemocytes after *in vivo* exposure to different cellular densities of *P. lima* for 24 h and 48 h. Control and PC represent negative and positive controls, respectively. The percentage of DNA in the comet tail is indicated by %tDNA. * indicates significant differences with respect to negative control in Mann-Whitney’s *U*-test (*p* < 0.05).

**Figure 3:**
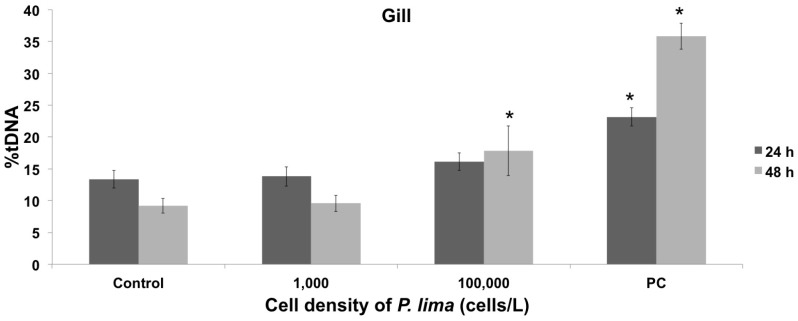
Quantification of DNA damage using the alkaline comet assay in mussel gill cells. Treatments and statistical analyses are as in Figure 2. * indicates significant differences with respect to negative control in Mann-Whitney’s *U*-test (*p* < 0.05).

Mussels were experimentally exposed to two cellular densities of the dinoflagellate *P. lima* (1,000 cells/L and 100,000 cells/L, for 24 h and 48 h, Figure 1). The subsequent accumulation of OA (the main DSP toxin) was used as an indicator of *P. lima* intake and the accumulation of DSP toxins by mussels, with results ranging between 21.67 ng/g and 112.12 ng/g dry weight (Table 1). Since these levels are well below the limit allowed by the European Commission Regulation for harvesting and sale (160 µg of OA equivalent/kg dry weight), mussel specimens were considered as being exposed to an early HAB stage and were used to evaluate the resulting genotoxic and cytotoxic effects. DNA damage was quantified in hemolymph and gill cells using the alkaline comet assay (Figure 1), with results showing a lack of significant genotoxic effects in both cell types at extremely low dinoflagellate concentrations (1,000 cells/L, after 24 h and 48 h exposure, Figure 2 and Figure 3). On the contrary, genotoxicity appeared to be dependent on the cell density of *P. lima* cultures, given the significant amount of DNA damage detected in hemolymph after a 24 h exposure to 100,000 cells/L (*p* < 0.05). These results are supported by the increase in the accumulation of OA by mussels exposed to higher *P. lima* concentrations (Table 1). On the contrary, no significant DNA damage was detected in gill cells after a 48 h exposure to 100,000 cells/L of *P. lima* (*p* < 0.05).

### 2.2. In Vivo vs. In Vitro Oxidative DNA Damage

**Figure 4:**
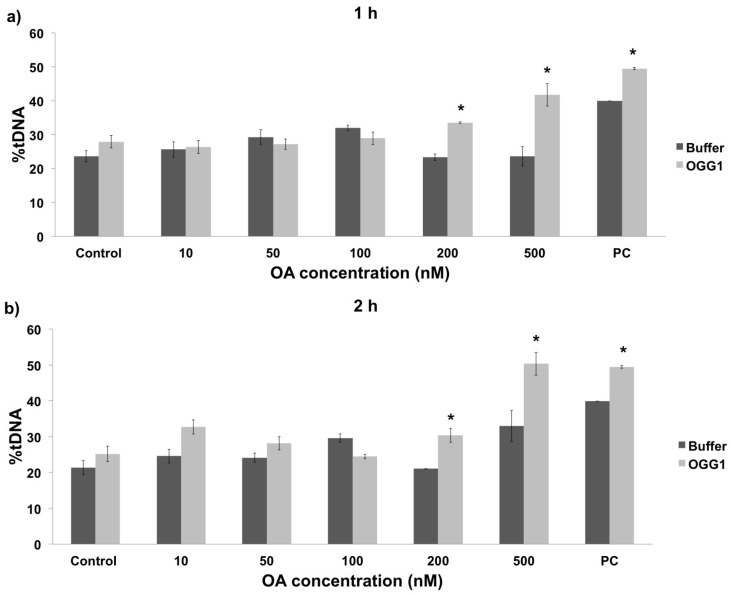
Quantification of oxidative DNA damage using the OGG1-modified comet assay in mussel hemocytes after *in vitro* exposure to different OA concentrations for 1 h (**a**) and 2 h (**b**). Control and PC represent negative and positive controls, respectively. The difference between buffer and OGG1 treatments specifically represents oxidative damage. The percentage of DNA in the comet tail is indicated by %tDNA. * indicates significant differences in respect to buffer in Mann-Whitney’s *U*-test (*p* < 0.05).

**Figure 5:**
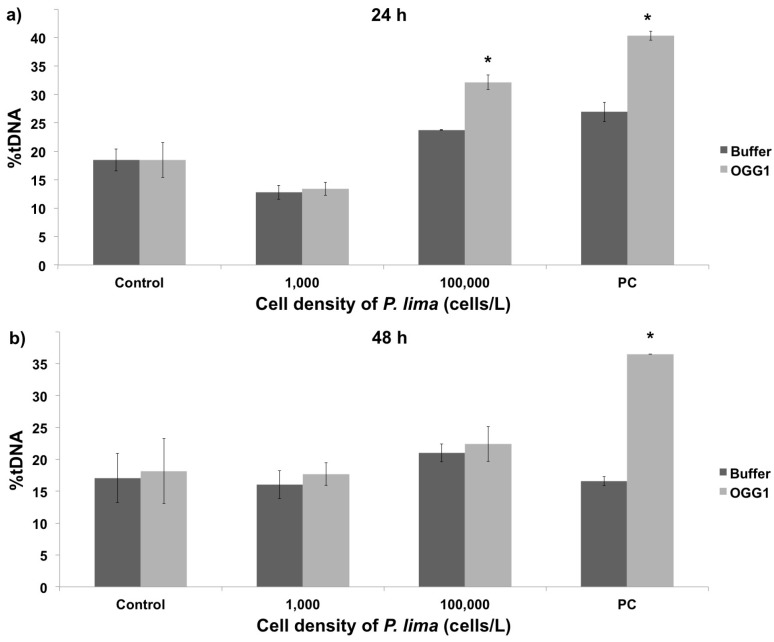
Quantification of oxidative DNA damage using the OGG1-modified comet assay in mussel hemocytes after *in vivo* exposure to different cellular densities of *P. lima* for 24 h (**a**) and 48 h (**b**). Controls and statistical analyses are as in Figure 4. * indicates significant differences in respect to buffer in Mann-Whitney’s *U*-test (*p* < 0.05).

Although the comet assay constitutes a useful method to assess the genotoxicity of the toxins produced by *P. lima*, the standard alkaline method provides limited information about the nature of the DNA damage. Therefore, this assay was modified with the enzyme OGG1 (a DNA-glycosysale responsible for the excision of 8-oxoGua) in order to study oxidative DNA damage, thereby allowing to specifically detect oxidized bases in the DNA [20]. For that purpose, the amount of DNA damage observed in the buffer treatment represents the overall damage resulting from single-stranded breaks (SSB), double-stranded breaks (DSB), and alkali-labile sites (ALS). On the other hand, the DNA damage observed in the presence of the OGG1 enzyme, corresponds to the combination of SSB, DSB, and ALS plus oxidative DNA damage. In the present work the modified comet assay was used to evaluate oxidative damage in hemolymph cells exposed *in vivo* to *P. lima* cultures. In addition, given the lack of *in vitro* data in this regard, the oxidative damage was also evaluated in cells directly exposed to purified OA. The obtained results showed that oxidative DNA damage remained constant in hemolymph cells exposed *in vitro* to low OA concentrations (10 to 100 nM) independently of exposure time, experiencing a significant increase at 200 nM and 500 nM (Figure 4, *p* < 0.05). The *in vivo* exposure to *P. lima* cultures revealed similar results, namely a significant increase in the percentage of oxidative DNA damage after exposure to high cell densities for 24 h (100,000 cells/L, Figure 5). However, the amount of oxidative damage seems to be dependent on exposure time in this case, as no significant effects were observed after 48 h exposure. Lastly, it must be noted that the results obtained without OGG1 are quite similar to those for the alkaline comet assay, supporting the complementarity of both approaches.

### 2.3. In Vivo Cytotoxic Damage After Exposure to P. lima

**Figure 6:**
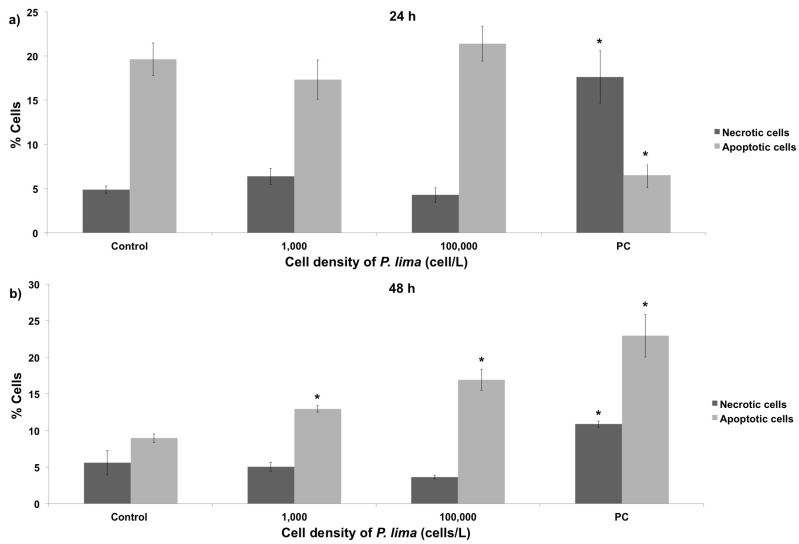
Flow cytometry evaluation of cytotoxicity in mussel hemocytes after *in vivo* exposure to different cellular densities of *P. lima* for 24 h (**a**) and 48 h (**b**). Control and PC represent negative and positive controls, respectively. The percentage of cells classified as necrotic or apoptotic is indicated by % Cells. * indicates significant differences in respect to negative control in Mann-Whitney’s *U*-test (*p* < 0.05).

The study of the genotoxic effects resulting from the *in vivo* exposure of mussel cells to the DSP-toxin producer *P. lima* was complemented with the characterization of cytotoxic damage using the Annexin assay (detection of phosphatidylserine in the outer leaflet of the plasma membrane [11,21]). This approach provides information about the apoptosis/necrosis rates, which can be subsequently compared with the DNA damage levels obtained in comet assays. Cell viability was evaluated on mussel hemocytes, revealing an absence of significant levels of cytotoxicity after 24 h exposure to low and high *P. lima* cell densities (Figure 6a). On the contrary, a significant increase in cytotoxicity was found after 48 h exposure (Figure 6b), underscoring the dependence of this type of damage mainly on the amount of exposure time. The differences between 24 h and 48 h negative apoptosis controls are probably the result of small variations during the preparation of samples for flow cytometry, which might include variation in the extraction of the cells, slight fluctuations in incubation temperatures, *etc.* In any case, these variations do not modify the major conclusions of the present analysis [22].

## 3. Discussion

In the present work, mussels were exposed *in vivo* to different cell densities of the DSP-producing dinoflagellate *P. lima*. With that in mind, OA concentration in tissue was used as an indicator of *P. lima* intake and accumulation of DSP toxins by experimental mussels. OA accumulation was subsequently compared with that present in mussels during natural HAB episodes, corroborating that the conditions used in this work mirror early stages of a HAB episode [23]. Although seafood is still fit for human consumption at this point, low toxin concentrations might encompass sublethal effects. The present work reveals that OA accumulation increases with time and, specially, with high cellular densities of *P. lima* (Table 1). Yet, the reduction found after a 48 h exposure to low *P. lima* concentrations (1,000 cells/L) might be mirroring an increase in the rate of depuration in mussels. On the contrary, exposure to higher *P. lima* concentrations would saturate depuration, accounting for the higher accumulation of OA observed at 100,000 cells/L. This is the first time that such response pattern has been detected in mussels, although steady OA concentration levels throughout time having been also described in the clam *Ruditapes decussatus* and in the mussel *Perna perna* after exposure to low cellular densities of *P. lima* [12,24]. Further studies will be required in order to clearly determine how depuration mechanisms affect toxin accumulation at low concentrations. It will be similarly interesting to ascertain how DSP estimation based on OA quantification from whole mussels (instead of independent tissues) might affect the results obtained here, especially as to how the effect of OA derivatives (notably 7-*O*-acyl derivatives or DTX3) might influence genotoxicity. Since toxin extractions were performed in the present work without an alkaline hydrolysis step before mass spectrometry (MS) quantification, the toxins analyzed included OA plus a partial extraction of DTXs. That raises the possibility that at least part of the genotoxic and cytotoxic effects observed in the present work could be attributed to contamination by the effect of DTX3 and OA acyl derivates (in addition to OA). That being said, it has been previously suggested that these derivates are formed at a very low rate during biotoxin exposure (at least for the case of *M. galloprovincialis*), with the bulk of their toxicity happening mostly at latter stages of prolonged DSP episodes [25]. Therefore, while the unaccounted effect of DTX3 and OA acyl derivates could be contributing to the genotoxicity/cytotoxicity determined in the present work, we suggest that such effects might be less important than previously anticipated, as the present work is focused on the early genotoxic and cytotoxic effects of *P. lima* exposure (*i.e.*, not enough time for the production of DTX3 and OA acyl derivates of significant amounts). Further studies will help elucidate the specific contribution of these compounds over time.

### 3.1. Mussel Responses to the Toxic Dinoflagellate P. lima

The results obtained in the present work revealed a conspicuous lack of genotoxic damage in mussel hemocytes, suggesting an absence of genotoxic stress at extremely low densities of *P. lima* [11,13,26]. Additionally, the resemblance in the DNA damage observed during *in vivo* exposure to *P. lima* and during *in vitro* exposure to OA might be indicative of a similar mode of toxin action in both cases [11,13,26]. Although previous studies have proposed that OA might have limited genotoxic potential [27] and that the chronic exposure to low-medium OA levels can lead to adaptation [28], our results support the ability of the toxins produced by *P. lima* (notably OA) to cause oxidative stress, similarly to what it has been described for mammalian cells exposed to low levels of other marine toxins [29,30]. Furthermore, it seems that mussels are able to repair DNA damage after short exposures (24 h) to low DSP and OA concentrations.

The analysis of the DNA damage induced indirectly by the exposure to *P. lima* in gill cells revealed a positive correlation with time and dinoflagellate concentration. However, while genotoxic effects were not observed after the first 24 h, an increase in DNA damage was evident after 48 h. Additional studies covering longer periods of time will be required to elucidate if resistance mechanisms are reinstated in mussel gills after longer exposure times, similarly to the case of clam gills after *in vivo* exposure to DSP toxins [11]. Altogether, similar amounts of DNA damage were found in hemocytes and gill cells, in agreement with *in vitro* results obtained for hemolymph and digestive glands of mussels and oysters exposed to OA [26]. However, hemocytes seem to experience genotoxic effects faster than gill cells, contrasting with previous *in vitro* results by our research group [13]. A discordance between the *in vivo* and *in vitro* effects of DSP toxins was also observed in gill cells, in agreement with previous results obtained in clams exposed to DSP toxins [11].

### 3.2. Oxidative DNA Damage in Mussel Hemolymph

The OGG1-modified comet assay was used in the present work to determine the amount of oxidative damage caused by *P. lima* on mussel DNA. Given that this assay cannot be implemented in gill cells due to the excessive basal DNA damage caused by OGG1 buffer, the results presented here refer to hemocytes. The comparison between *in vivo* exposure to *P. lima* and *in vitro* exposure to OA revealed that this biotoxin causes oxidative damage both directly and indirectly on this cell type. More specifically, DNA damage appears to be time-independent after 2 h exposure under *in vitro* conditions. On the contrary, a significant decrease in the level of oxidative damage was observed after a 48 h *in vivo* exposure. A similar reduction in damage after longer exposure times was previously described in molluscs [31,32,33,34]. While these results are consistent with a higher oxidative genotoxic potential of OA *in vitro*, it is also evident that exposure to *P. lima* can induce oxidative DNA damage in hemocytes mainly as a consequence of Reactive Oxygen Species (ROS) formation, although that is most likely alleviated by antioxidant mechanisms *in vivo*.

The results from modified comet experiments revealed a significant increase in oxidative DNA damage at high densities of *P. lima*, very early (24 h exposure) during HAB simulation. This effect is concurrent with closure periods in commercial mussel rafts, probably due to saturation in depuration mechanisms and lower activity of cellular antioxidant defenses. Different studies have provided evidences of oxidative stress caused by DSP toxins in marine organisms, notably by evaluating transcriptional levels of genes encoding detoxifying enzymes and biochemical markers [16,34]. In those cases, an increase in the effect of antioxidant mechanisms was observed, concomitantly with the accumulation of DSP toxins [34]. Additional transcriptomic analyses (together with other omic approaches) of mussels exposed to DSP toxins will be critical to elucidate the mechanisms by which these toxins affect marine invertebrates *in vivo*. Overall, the differences observed between *in vitro* and *in vivo* studies are in agreement with previous studies [35], where the synergistic interactions between OA and other DSP toxins might be responsible for higher levels of toxicity [3,36] and/or different toxicological effects [37].

### 3.3. Cytotoxic Damage in Mussel Hemocytes

Flow cytometry results did not show significant differences between necrosis and apoptosis levels in mussel hemocytes exposed *in vivo* to *P. lima* for 24 h. Apoptosis, on the contrary, increased significantly after 48 h, supporting results from our previous work studying the *in vitro* effect of OA in mussels [13]. By comparing cytotoxicity data, OA accumulation at different exposure times and *P. lima* concentrations (Table 1), it appears that exposure to *P. lima* does not cause significant cytotoxic damage at intermediate concentrations, supporting previous results obtained in mussels exposed *in vitro* to OA [23]. However, the opposite situation is observed at extreme *P. lima* densities, encompassing a significant increase in the percentage of apoptotic cells. The discordance between this observation and the aforementioned *in vitro* reports [23] might be determined by the use of different types of controls. Accordingly, while *in vitro* exposures used control specimens with very low concentrations of OA (48 ng/g dry weight), the present work used control mussels virtually lacking any OA (<0.10 ng/g). Indeed, a similar increase in the number of apoptotic cells over time was found on hemocytes from carpet shell clams exposed to OA, using controls completely lacking OA [24]. Lastly, the increase in the number of apoptotic cells observed in the present work is negatively correlated with the number of necrotic cells, displaying a stable number of viable cells. This result is similar to those reported by several other papers, suggesting that OA does not have a negative influence in the viability of mussel cells [13,24,38,39,40] probably due to a higher number of protein apoptosis inhibitors (IAPs) in bivalve molluscs [38] compared with other species.

## 4. Conclusions

This study is the first describing the early genotoxic and cytotoxic effects resulting from the *in vivo* exposure of the mussel *M. galloprovincialis* to the DSP-toxin-producing dinoflagellate *P. lima*. The obtained results revealed that: (1) low *P. lima* cell densities can be used to recreate early stages of HAB episodes in laboratory conditions; (2) the *in vivo* exposure to extremely low dinoflagellate concentrations (1,000 cells/L) did not produce significant genotoxic effects in hemocytes or gill cells; (3) the DNA damage observed in gill cells exposed *in vivo* to *P. lima* was predominantly influenced by exposure time and *P. lima* cell density; (4) the oxidative DNA damage of hemocytes exposed *in vitro* to OA was dependent on toxin concentration, while in the case of hemocytes exposed *in vivo* to *P. lima* damage was only observed after 24 h exposure to the highest dinoflagellate concentration studied (100,000 cells/L). This suggests a more relevant role of the antioxidant system in this latter case; (5) the absence of significant levels of DNA damage at low *P. lima* and OA concentrations underscores the inability of these toxin concentrations to disturb the antioxidative balance in mussel cells; (6) *in vivo* exposure to growing *P. lima* densities increased apoptosis but not necrosis, probably due to a high number of protein apoptosis inhibitors (IAPs) in molluscs. Overall, these conclusions increase the knowledge regarding the *in vivo* genotoxic and cytotoxic potential of the marine biotoxins produced by the dinoflagellate *P. lima* in bivalve molluscs. In doing so, this work demonstrates for the first time the suitability of the modified (OGG1) comet assay as a valid experimental approach improving the evaluation of the oxidative DNA damage caused by marine toxins in the hemolymph of marine invertebrates. Furthermore, the present work corroborates the value of the comet assay and flow cytometry as a means to evaluate early genotoxic and cytotoxic responses, laying the foundations for future experiments aimed at identifying exposure biomarkers and mitigating economic losses associated with HABs in coastal areas.

## 5. Materials and Methods

### 5.1. Specimen Collection and Microalgae Cultures

*M. galloprovincialis* individuals (5–7 cm shell length) were obtained from a commercial mussels raft from Lorbe in the Ria of Ares-Betanzos (Galicia, NW Spain) in April 2015 (Figure 1).The invertebrate animals experiment was assessed by the Spanish Ministry of Economy and Competitivity (project AGL2012-30897 and approved on 28 December 2012.). These rafts (previously used in our research [13]) were chosen as sampling sites based on the low density of toxic microalgae [41]. Mussels were acclimated to laboratory conditions (18 °C, 12 h light-dark cycle) for a week in vigorously aerated tanks with filtered sea water, and fed two times a day with a 1:1 mixture of two nontoxic microalgae species (*Isochrysis galbana* and *Tetraselmis suecica*). The culture of the DSP-toxin-producing dinoflagellate *P. lima* (strain AND-A0605) was obtained from the Quality Control Laboratory of Fishery Resources (Huelva, Spain). The production of OA (the main DSP toxin) in *P. lima cultures* was quantified as 0.4 pg OA/cell using High Performance Liquid Chromatography/Mass Spectrometry (HPLC/MS). Based on this data, mussels were exposed to two different cell densities (1,000 and 100,000 cells/L) of toxic dinoflagellate for 24 h and 48 h, simulating OA concentrations observed during the early stages of the development of an algal bloom [1]. Cell concentrations in the *P. lima* culture were determined by cell count in Sedgwick-Rafter counting slides (Pyser-Sgi, Edenbridge, UK) after fixation with Lugol´s solution.

### 5.2. Sample Preparation and HPLC/MS Analysis

HPLC/MS analyses were carried out by the chromatography unit at SAI-University of A Coruña, following the protocol of the European Union Reference Laboratory for Marine Biotoxins [42]. Toxin extractions were performed without alkaline hydrolysis before MS, consequently, these included complete extraction of OA, plus partial extraction of DTXs. For that purpose, certified Reference Material Mussel Tissues with certified values of 10.1 ± 0.8 µg OA/g and 1.3 ± 0.2 µg DTX1/g (NRC CRM-DSP-Mus-b; Institute for Marine Biosciences, National Research Council of Canada, Halifax, NS, Canada) were used for recovery determination in the HPLC/MS method. Accordingly, mussel tissues (20 g per sample) were lyophilized (Christ LMC-2, model beta 2–16, Christ, Osterode, Germany) and 2 g of the liophilizate were extracted three times with 15 mL of 100% methanol and homogenized for 1 min. The methanolic phase was subsequently centrifuged (5000 *g* for 25 min) and the supernatant was filtered (0.45 µm pore size) and transferred to a 50 mL volumetric flask. HPLC/MS analyses were performed on a Thermo LTQ Orbitrap instrument (Thermo Fisher Scientific, Bremen, Germany), using a C18 column (5 mm, 150 mm × 4.6 mm) at 30 °C (Phenomenex, Aschaffenburg, Germany). The mobile phase A was 100% water with 16 mM ammonium formate, and the mobile phase B was 100% acetonitrile. Gradient elution from 30% to 90% B was performed over 6 min; then, 90% B and 10% A were held for 6 min, decreased to 30% B over 4 min, which was held again for 10 min until the next run. The flow rate was set at 0.5 mL/min using an injection volume of 20 µL. Detection was performed using electrospray ionization (ESI) coupled with multiple reaction-monitoring (MRM). The electrospray capillary was set at 4 kV, the nebulizer at 50 arbitrary units, dry gas at 50 arbitrary units, and dry temperature at 350 °C. Data analyses and peak integration were accomplished through the ThermoXcalibur™ software (Thermo Fisher Scientific, San José, CA, USA).

### 5.3. In Vivo Exposure to P. lima

After acclimation, mussels were randomly divided into three groups (Figure 1) including: a control group fed with a 1:1 mixture of the microalgae *I. galbana* and *T. suecica*, and two experimental groups fed with the DSP toxin-producing *P. lima* (1,000 and 100,000 cells/L, respectively, four times a day). Total OA body burden was determined in tissue homogenates after exposures (indirect measure of *P. lima* intake by mussels) according to the procedure described above. Mussel specimens were exposed to *P. lima* cultures in groups of *n* = 25 individuals (three replicates). Experimental samples were randomly sampled from tanks to complete a total of 20 g of pooled mussel tissue (using approximately *n* = 5–8 individuals), which was subsequently lyophilized. Analyses were performed from a sample (2 g) of each lyophilized fraction. Complementary to *in vivo* exposures, the assessment of oxidative DNA damage was completed using the modified comet assay on hemolymph pools from 20 control individuals exposed *in vitro* to OA (Sigma-Aldrich, St. Louis, MO, USA) for 1 h and 2 h (these conditions were selected as representative of early genotoxic effects of OA based on our previous analyses [13]). For that purpose, OA was diluted in dimethyl sulfoxide (10, 50, 100, 200, and 500 nM final concentration) and 10 µL of each OA solution was added to cell suspensions (15 °C to 18 °C). Exposures were completed by pelleting cells (825 g for 3 min) before proceeding with the comet assay. *In vitro* incubations with hydrogen peroxide (100 µM for 10 min and 100 µM for 2 min) or camptothecin (4 µM for 4 h) were used as positive control in the comet assay, modified comet assay, and flow cytometry experiments, respectively.

### 5.4. Isolation of Hemocytes and Gill Cells

Hemolymph and gill cells were used to quantify DNA damage using the alkaline comet assay. Additionally, hemolymph cells were used to assess oxidative DNA damage and cytotoxicity by means of the modified comet assay and flow cytometry, respectively (Figure 1). Hemolymph samples from each mussel (1.5 mL) were withdrawn from the posterior adductor muscle with a sterilized syringe and were mixed simultaneously (1:5) with a precooled modified Alsever´s anticoagulant solution (NaCl 382 mM, glucose 115 mM, sodium citrate 27 mM, EDTA 11.5 mM). The hemolymph from five mussels was pooled to eliminate interindividual variation and was subsequently filtered using a 55 µm nylon mesh, counting the number of hemocytes in a Thoma chamber (Marienfeld, Lauda-Königshofen, Germany) under the microscope. Gill cells from the same five mussels were isolated as described elsewhere [13,43], dissected at room temperature, and washed three times in 2 mL of ice-cold calcium magnesium-free saline solution (CMFS: 20 nM HEPES, 500 mM NaCl, 12.5 mM KCl, 5 mM EDTA in RPMI medium). Gills were then meticulously shredded and the resulting suspension was placed in a tube containing 4 mL of CMFS and shaken gently for 1 h at 4 °C in the dark. The entire suspensions were then filtered gently (55 µm nylon mesh) and centrifuged at 500 *g* for 5 min. The resulting pellet was re-suspended in 1 mL of Kenny´s Salt Solution (KSS: 0.4 M NaCl, 9 mM KCl, 0.7 mM K_2_HPO_4_, 2 mM NaHCO_3_) and kept on ice until required. Gill cell number was determined microscopically. Cell viability was determined by trypan blue exclusion method, setting the viability threshold at 80% in all samples used.

### 5.5. In Vitro Exposure to OA

The oxidative DNA damage produced by direct *in vitro* exposure to OA was evaluated in hemolymph cells using the modified (OGG1) comet assay. Accordingly, hemolymph was pooled from 20 mussels belonging to the control group and subsequently exposed *in vitro* to OA (Sigma-Aldrich, St. Louis, MO, USA) for 1 and 2 h (the range of OA concentrations and exposure times were selected based on previous reports [13]). OA was diluted in DMSO to final concentrations of 10 nM, 50 nM, 100 nM, 200 nM, and 500 nM, adding 10 µL to the cell suspensions (15 °C to 18 °C). Exposures were stopped by centrifugation (825 *g* for 3 min), collecting the pelleted cells for comet assay. *In vitro* incubations with hydrogen peroxide (100 µM, 10 min; 100 µM, 2 min) or camptothecin (4 µM, 4 h) were used as positive control in the comet assay, the modified comet assay and flow cytometry experiments, respectively.

### 5.6. Alkaline Comet Assay

The alkaline comet assay was performed as described elsewhere [13,44] with slight modifications as follows. Hemolymph cells were centrifuged for 5 min at 250 *g*, and gill cells were centrifuged for 3 min at 1,000 *g*. The resulting pellets were re-suspended in 90 μL of 0.5% low-melting-point agarose (Invitrogen, Carlsbad, CA, USA) in KSS. Each sample was divided in two, placed on a slide pre-coated with a layer of 0.5% normal-melting-point agarose (Intron Biotechnology, Gyeonggi-do, Korea), and incubated at 4 °C for 25 min. The slides were subsequently placed in a Coplin jar with lysis solution (2.5 M NaCl, 100 mM Na_2_EDTA, 250 mM NaOH, 10 mM Tris-HCl, 1% sarcosyl, pH 10 with 1% Triton X-100 added just before use) for 1 h at 4 °C. From this point on, all steps were conducted in the dark to prevent additional DNA damage. After lysis, slides were placed in an alkaline solution (0.3 M NaOH, 1 mM Na_2_EDTA, pH > 13) for 20 min for DNA unwinding, and subjected to electrophoresis for 20 min (0.83 V/cm). Slides were subsequently washed with cold neutralization buffer (0.4 M Tris-HCl, pH 7.5), stained with 4,6-diamidino-2-phenylindole (DAPI), and stored in the dark at 4 °C. Image capture and analysis was performed using the Comet IV Software (Perceptive Instruments, Bury St Edmunds, UK). Fifty cells were scored from each replicate slide (100 cells total) and the percentage of DNA in the tail (%tDNA) was used as DNA damage parameter.

### 5.7. Modified Comet Assay with OGG1 Incubation

A modified version of the comet assay incorporating incubation with the 8-oxoguanine DNA glycosylase (OGG1) repair enzyme was used to assess the oxidative DNA damage in hemolymph cells (the excessive basal damage in gill cells precluded its inclusion in this analysis), according to the procedure described in [20]. After the lysis treatment, slides reserved for the modified comet assay were washed three times for 5 min in a Coplin jar with OGG1 buffer (40 mM HEPES, 100 mM KCl, 0.5 mM EDTA, 0.2 mg/mL BSA, pH 8). Fifty microliters of OGG1 enzyme (0.0016 U/µL, New England Biolabs, Beverly, MA, USA) were added to each slide and covered with a slip (controls were treated with 50 µL of buffer without enzyme). After incubation at 37 °C in a dark, humidified chamber for 10 min, the cover slips were removed and the slides were placed on an electrophoresis platform with the rest of slides, following subsequent steps similar to the alkaline comet assay.

### 5.8. Flow Cytometry Cytotoxicity Assay

The apoptosis/necrosis induced by OA (used as indicator of the effect of *P. lima* exposure) was evaluated by means of flow cytometry using Annexin V-Phycoerythrin (PE) and 7-Amino-Actinomycin (7-AAD) staining. The Annexin V-PE Apoptosis Detection Kit I (BD Biosciences, Franklin Lakes, NJ, USA) was used according to the manufacturer´s guideline with minor modifications. Gills cannot be used in this approach due to the presence of aggregated cells and debris. After OA exposure, hemolymph samples were centrifuged at 250 *g* for 5 min at 4 °C. The cell pellet was re-suspended in 200 µL of annexin binding buffer (0.5×) previously diluted in saline solution (NaCl, 500 nM) to a final concentration ranging between 4 × 10^5^ and 6 × 10^5^ cells. Annexin V-PE and 7-AAD (1 mg/mL) were added and incubated for 15 min at room temperature in the dark before analyzing cells by flow cytometry. The hemocyte population was fixed in the dot-plot following [23] and analyses were carried out in a FACScalibur flow cytometer (BD Biosciences, Franklin Lakes, NJ, USA). A minimum of 20,000 events were acquired in each case and fluorescence signals for Annexin V-PE and 7-AAD were measured using the FL-2 and FL-3 detectors, respectively. The percent of apoptotic and necrotic cells were analyzed using Cell Quest Pro software (BD Biosciences, Franklin Lakes, NJ, USA). Early apoptosis and late apoptosis/necrosis were expressed as the mean ± SE percentages of annexin V+/7-AAD- and annexin V+/7-AAD+ cells, respectively.

### 5.9. Statistical Analyses

At least three replicates were performed for each experimental condition tested. Experimental data were expressed as mean ± standard error and were tested for normality using the Kolmogorov-Smirnov test. As the data obtained showed a violation of the assumption of normality, non-parametric testing was deemed adequate. Differences between groups were therefore tested using Kruskal-Wallis and Mann-Whitney *U*-tests. *p*-values < 0.05 were considered significant. Statistical analyses were performed using the IBM SPSS software package V. 20 (IBM, Armon, NY, USA).

## Figures and Tables

**Table 1:** The intake of *P. lima* by mussels after *in vivo* exposures was estimated by quantifying the accumulation of okadaic acid. Data resulting from three independent experimental replicates.

*P. lima* (Cell/L)	Exposure Time (h)	Mean OA (ng/g Dry Weight) ± Standard Error
1,000	24	28.35 ± 3.07
1,000	48	21.67 ± 2.02
100,000	24	64.77 ± 5.77
100,000	48	112.12 ± 7.78

## Abbreviations

The following abbreviations are used in this manuscript:

ALS: Alkali Labile Site
DSB: Double-Stranded Breaks
HAB: Harmful Algal Bloom
HPLC/MS: High Performance Liquid Chromatography/Mass Spectrometry
IAP: Apoptosis Inhibitory Proteins
OA: Okadaic Acid
OGG1: 8-oxoguanine DNA glycosylase
SSB: Single-Stranded Breaks
